# Aberrant Oscillatory Activity during Simple Movement in Task-Specific Focal Hand Dystonia

**DOI:** 10.3389/fneur.2012.00165

**Published:** 2012-11-28

**Authors:** Leighton B. N. Hinkley, Rebecca Dolberg, Susanne Honma, Anne Findlay, Nancy N. Byl, Srikantan S. Nagarajan

**Affiliations:** ^1^Department of Radiology and Biomedical Imaging, University of CaliforniaSan Francisco, CA, USA; ^2^Department of Physical Therapy and Rehabilitation Science, University of CaliforniaSan Francisco, CA, USA

**Keywords:** focal hand dystonia, neuroimaging, magnetoencephalography, motor cortex

## Abstract

In task-specific focal hand dystonia (tspFHD), the temporal dynamics of cortical activity in the motor system and how these processes are related to impairments in sensory and motor function are poorly understood. Here, we use time-frequency reconstructions of magnetoencephalographic (MEG) data to elaborate the temporal and spatial characteristics of cortical activity during movement. A self-paced finger tapping task during MEG recording was performed by 11 patients with tspFHD and 11 matched healthy controls. In both groups robust changes in beta (12–30 Hz) and high gamma (65–90 Hz) oscillatory activity were identified over sensory and motor cortices during button press. A significant decrease [*p* < 0.05, 1% False Discovery Rate (FDR) corrected] in high gamma power during movements of the affected hand was identified over ipsilateral sensorimotor cortex in the period prior to (−575 ms) and following (725 ms) button press. Furthermore, an increase (*p* < 0.05, 1% FDR corrected) in beta power suppression following movement of the affected hand was identified over visual cortex in patients with tspFHD. For movements of the unaffected hand, a significant (*p* < 0.05, 1% FDR corrected) increase in beta power suppression was identified over secondary somatosensory cortex (S2) in the period following button press in patients with tspFHD. Oscillatory activity within in the tspFHD group was however not correlated with clinical measures. Understanding these aberrant oscillatory dynamics can provide the groundwork for interventions that focus on modulating the timing of this activity.

## Introduction

Task-specific focal hand dystonia (tspFHD) is a debilitating movement disorder characterized by involuntary co-contractions of agonist and antagonist muscles of the hand and forearm during specific, well-learned activities such as playing a musical instrument, writing, or typing (Fahn et al., 1998). While the etiology of tspFHD is considered idiopathic, most researchers and clinicians agree focal dystonia is a multifactorial disorder developing from an interaction of both extrinsic (e.g., trauma, injury to the upper extremity, neuropathy, stress, perfectionism, poor ergonomics and/or repetitive overuse; Jankovic, 2001; Jabusch et al., 2004), and intrinsic factors (e.g., genetics, musculoskeletal limitations, neurophysiological abnormalities, personality; Gasser et al., 1998; Breakefield et al., 2008). It is not clear whether intrinsic factors predispose individuals to developing tspFHD or whether the observed aberrations in intrinsic features represent the consequences of the disorder.

There is emerging evidence from non-invasive neuroimaging studies that processing within both the sensory and the motor system are abnormal in tspFHD. Aberrant cortical sensory topographical organization has been reported in animal and human subjects with tspFHD (Byl et al., 1996, 1997; Meunier et al., 2001). Levels of cortical motor activity identified using modalities like fMRI and PET are also known to be outside the normal range for patients with dystonia compared to age matched controls. For example, cortical fields involved in the preparation and execution of self-initiated movements in the contralateral hemisphere are known to be both hyperactive (in the case of primary motor cortex; M1) and hypoactive [in premotor cortex (PMC); Ibanez et al., 1999; Lerner et al., 2004; Obermann et al., 2008]. In the primate neocortex, M1 and PMC are densely interconnected across the two hemispheres (Boussaoud et al., 2005) and communicate with each other on a millisecond time scale (Rubino et al., 2006). Brain mapping modalities that interrogate cerebral blood flow can only provide a static image of activity during a motor behavior, and lack the temporal fidelity to track changes in the brain that occur during discrete stages of movement.

The primary goal of this study was to characterize the timing, amplitude, and duration of activity across the motor cortices bilaterally in patients with tspFHD using novel time-frequency decompositions of magnetoencephalography (MEG) data. Typically, a significant decrease in beta oscillations (12–30 Hz) and increase in high gamma synchrony (>30 Hz) localizes to the contralateral motor strip both preceding (∼200 ms) and following movements of the hand (Taniguchi et al., 2000; Pfurtscheller et al., 2003; Jurkiewicz et al., 2006; Cheyne et al., 2008; Dalal et al., 2008; Muthukumaraswamy, 2010). Since even basic temporal processing in the motor system has not been studied to date in tspFHD, we chose a simple, self-paced finger movement to investigate oscillatory fluctuations in M1 and PMC across the two hemispheres. Similar to previous studies (e.g., Obermann et al., 2008) we chose a task that would not induce dystonic posturing in order to avoid experimental confounds (e.g., EMG noise, sustained contractions during movement), We hypothesized that aberrant levels of motor activity in tspFHD would manifest prior to and during movement in the beta and high gamma frequency bands. The secondary goal of this study was to determine if clinical parameters of function, sensory discrimination, fine motor speed, strength, and motor control could be used to predict aberrant beta and high gamma activity in the motor cortices. Individuals with tspFHD demonstrate clinical deficits in stereognosis and graphesthesia and intrinsic muscle strength in the hand in addition to reduced quality of motor performance at a dystonic task (Byl et al., 2002; McKenzie et al., 2003, 2009). We hypothesized that these behavioral deficits were correlated to changes in oscillatory activity in M1 during hand movement.

## Materials and Methods

### Subjects

Eleven right handed subjects with tspFHD (Table 1) were recruited to participate in the study through the Faculty Practice in Physical Therapy and the Movement Disorders Clinic at UCSF. Only right hand patients were included because it is thought that handedness may affect the motor paradigm. Subjects were clinically evaluated for severity using the arm subscore of the Fahn–Mardsen Dystonia Movement Scale (FMDMS; Burke et al., 1985; Fahn et al., 1987). Inclusion criteria included: ages 21–75 years, a ranking of 2–3 on the arm subscore of the FMDMS (indicating moderate or severe motor impairment), clear dystonic movements during performance of a dystonic task, and no botulinum toxin A injections 3 months prior to participation. Exclusion criteria included: other systemic or neurologic disease associated with a known movement disorder, presence of tremor, medical instability, or MEG/MRI contraindications (e.g., pacemakers, metallic implants). Participants were carefully selected to only include those with one type of focal dystonia (e.g., excluding participants with blepharospam) and to exclude those whose dystonia extended beyond the hand (e.g., arm).

**Table 1:** **Description of subjects with task-specific focal hand dystonia**.

Patient	Affected hand	Dominant hand	Target task	Gender (M/F)	Age(years)	Most affected digit	Severity	Treatment
1	Right	Right	Writing	M	53	D3	Severe	None
2	Right	Right	Writing	M	41	D2	Severe	None
3	Right	Right	Guitar	M	48	D3	Severe	One botulinum toxin A injection; did not help
4	Right	Right	Typing	M	43	D2	Severe	None
5	Right	Right	Writing	F	46	D2	Moderate	None
6	Right	Right	Writing/typing	M	27	D2	Moderate	None
7	Right	Right	Drums	M	53	D2	Severe	One botulinum toxin A injection; did not help
8	Right	Right	Writing/typing	M	36	D2	Moderate	None
9	Right	Right	Writing	F	40	D2	Severe	None
10	Right	Right	Guitar	M	32	D2	Severe	None
11	Right	Right	Writing	F	43	D2	Moderate	One botulinum toxin A injection; did not help
Healthy controls	N/A	11R	N/A	8M/3F	24–66	N/A	N/A	

There were three females and eight males, with a mean age ± SD, 42.0 ± 8.1 years. Seven of these patients had severe dystonia (Arm FMDMS = 2), four with moderate dystonia (Arm FMDMS = 3; Table 1). Eleven right handed volunteers (eight males, three females; mean age ± SD, 38 ± 14.6 years) served as healthy controls. The healthy control participants were matched to gender in the tspFHD group (three females in either cohort). Healthy control participants recruited for this study were also matched for age in order to eliminate any age-related changes in beta or high gamma oscillatory power (Wilson et al., 2010), with no significant difference in age between the healthy control and patient groups (*p* > 0.05). All recruited subjects with focal hand dystonia and healthy volunteers gave written consent for the study prior to participation. This study was approved by the Committee on Human Research of the University of California, San Francisco, USA.

### Data acquisition and task

Magnetoencephalographic data was acquired with a 275-channel CTF whole-head MEG system (MISL; Coquitlam, BC, Canada) with a 1200 Hz sampling rate. Head position relative to the MEG sensors was determined with fiducial coils (nasion, right, and left preauricular points). For source space reconstructions, T1-weighted structural MR images were obtained for each subject using a 1.5T MRI scanner (GE Medical System, Milwaukee, WI, USA; flip angle = 40°, TR/TE = 27/6 ms, FOV = 240 × 240 mm, 1.5 mm slice thickness, 256 × 256 × 124 pixels).

A self-paced button press protocol was used to investigate a self-initiated movement of a digit using a procedure identical to previous studies known to produce changes in neural activity within the cortical and sub-cortical motor system during voluntary motor behavior (Rektor et al., 2001; Sochurkova et al., 2006; Dalal et al., 2008) Patterns of induced activity produced using this paradigm are similar to those elicited when movement is behaviorally linked with an external sensory stimulus (Bares and Rektor, 2001; Rektor et al., 2003). Subjects were instructed to depress a MEG-compatible button approximately once every 3 s at a self-paced interval for ∼100 events. The most affected digit was selected for the subjects with focal hand dystonia as well as the corresponding digit on the unaffected hand. Digit two of the right and left hand was selected for healthy volunteers.

The self-paced button press task was designed to be simple enough that it would eliminate the possibility of any aberrant motor behaviors during data collection, including dystonic cramping and mirror movements with the ipsilateral hand. In addition, several independent measures were in place in order to monitor participant hand position. First, participants were asked to execute a few trial movements of the button press task prior to MEG recording in order to make sure that the movements did not produce discomfort. Second, all movements were monitored during the scan session through a video monitor in the magnetically shielded room by a trained specialist in movement disorders (Rebecca Dolberg, Nancy N. Byl). Criteria used to diagnose focal hand dystonia through video analysis (Byl, 2004) were applied to video observations of the scan session in order to verify that no dystonic movements occurred during data collection. Finally, the onset and duration of the response (button press) was recorded through a set of audio-to-digital output channels from the response button. Across both groups, all participants reported being comfortable during data collection, and there were no reports of cramping or dystonic posturing during the task. In addition, no hand cramping or mirror movements were observed by the independent raters through the video monitor. Button press duration did not differ (*p* = 0.21) between healthy controls (mean = 290.7 ms, SD = 171.6 ms) and movements of the affected hand in the tspFHD group (mean = 413.1 ms, SD = 261.6 ms).

### Clinical performance measures

All subjects with focal hand dystonia were evaluated using a battery of clinical measures for function, sensory discrimination, motor speed, strength, and task-specific motor control (Byl et al., 2002, 2003; McKenzie et al., 2003, 2009). Overall level of function was assessed using the Café 40, a questionnaire that evaluates activities of daily living (Fung et al., 1997). Sensation was assessed using graphesthesia (modified Jean Ayers Sensory Integration Praxis Test) and stereognosis (Byl–Cheney–Boczai Sensory Discriminator; Byl et al., 2002). These scores were converted to indicate the percentage of correct answers given and combined to form a sum sensory discrimination score. Motor speed and accuracy was assessed using a stopwatch (digital reaction time; Bohannon, 1995), and a finger tapper (PAR Psychological Assessment; Dilks, 2006). A sum motor speed score was then calculated by summing the normative score of the digital reaction time test with the average score from the tapping speed test. Grip strength was measured with a handheld Jamar dynamometer (Peolsson et al., 2001). Pinch strength was measured using a three jaw chuck pinch and lateral pinch strength was measured with a pinch dynamometer (Peolsson et al., 2001; Byl et al., 2003). Lumbrical strength was measured with a MicroFet dynamometer (Byl et al., 2003). A sum score was then calculated for strength by summing the averages for each of the strength measures.

Task-specific motor control was assessed using video analysis developed by Byl et al. (2002). Subjects were asked to perform the target task and graded on an ordinal scale for posture, movement patterns, and control of movement. A percent score of the total possible points was then calculated. A score of 100% would indicate no problems in movement control.

### Imaging data analysis

Prior to analysis, artifacts (e.g., eyeblink, head movement) in the MEG sensor data were manually removed by detecting events with excessive noise (signal amplitude > 10 pT). MEG sensor data were reconstructed spatiotemporally in the time-frequency domain using an adaptive spatial filtering technique (Dalal et al., 2008; Hinkley et al., 2011) implemented in NUTMEG^1^ using the shared computing cluster at the California Institute for Quantitative Biomedical Research^2^. These time-frequency reconstructions model induced, non-phase locked changes in oscillatory activity (Dalal et al., 2008; Hinkley et al., 2011). Tomographic volumes of possible source locations (voxels; lead field resolution = 5 mm) were computed through an adaptive spatial filter which weights each source location relative to the signal of the MEG sensors (Dalal et al., 2008). Source power for each location is estimated using a noise-corrected pseudo-F statistic contrasting a sliding experimental (active) time window to a fixed baseline (inter-trial) period of the same length, and are expressed in logarithmic units (decibels, dB; see Robinson and Vrba, 1999; Dalal et al., 2008). Active time windows consisted of a sliding (50 ms step size) window across the theta/alpha (4–12 Hz; 300 ms windows), beta (12–30 Hz; 200 ms windows), gamma (30–55 Hz; 150 ms windows), high gamma, and ultra-high gamma (65–90 Hz, 90–115 Hz; 100 ms windows) frequency bands at widths optimized for time-frequency reconstruction in source space (Dalal et al., 2008; Hinkley et al., 2011).

For the whole brain group analysis of the time-frequency data, individual statistical maps were spatially aligned by applying a transformation matrix derived from the normalization of the subject’s anatomical T1 to the beamformer volume (Dalal et al., 2008; Hinkley et al., 2011). Both within- and between-subject group analyses were performed using non-parametric permutation testing (SnPM; Singh et al., 2003). Average and variance maps for each individual time window were calculated and smoothed using a 20 mm^3^ FWHM Gaussian kernel (Barnes et al., 2004; Guggisberg et al., 2010). Statistical maps were corrected for multiple corrections using a false discovery rate (FDR) of 1% at a significance threshold set at *p* ≤ 0.05 obtained from the non-parametric randomization test. Following statistical thresholding, the location of sources (peak power change in a cluster) in MNI space were converted to Talairach coordinates(mni2tal^3^) and anatomical labels were defined using the Talairach Daemon database^4^.

As part of an exploratory analysis designed to test the hypothesis that aberrant motor activity is related to behavioral impairments in tspFHD, MEG beta oscillatory power over contra- and ipsilateral M1 was correlated with clinical performance measures in the focal hand dystonia group using a Pearson product-moment correlation coefficient. A feedforward stepwise linear regression analysis was also performed to determine if clinical parameters could predict either the latency or amplitude of an aberrant motor response. The affected hand and unaffected hand of subjects with focal hand dystonia were analyzed separately. For the correlation analyses, we only focused on both the amplitude and peak latency of M1 activity in each group.

## Results

### Whole brain analyses of motor activity

#### Within-group analyses

**Figure 1:**
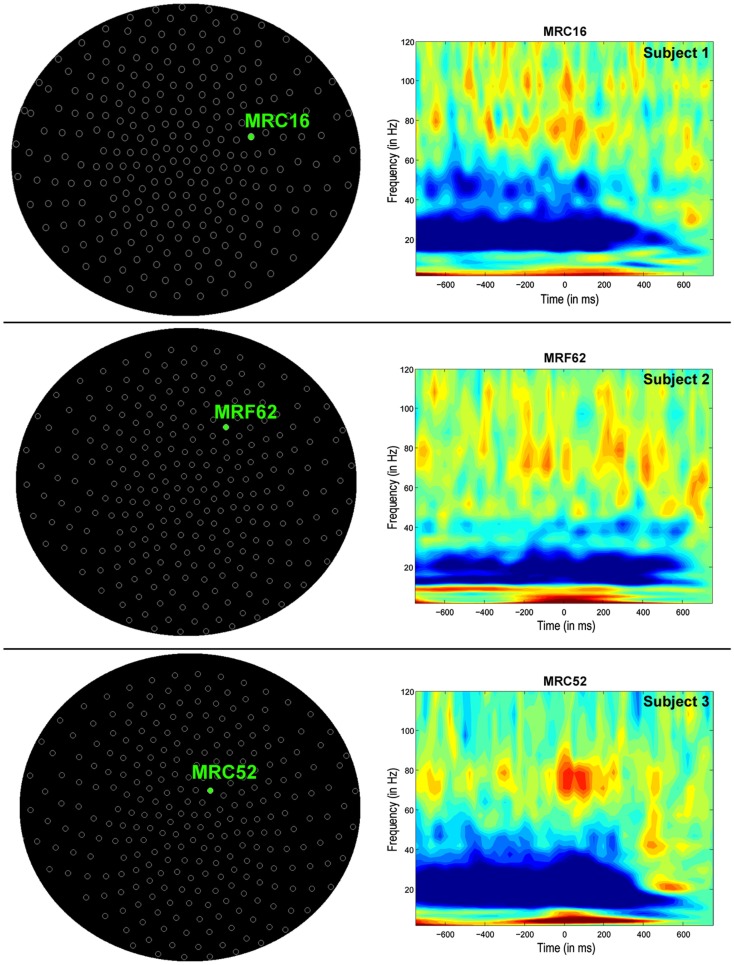
**Sensor-level analyses of MEG motor data in three exemplary healthy control participants during movements of the left hand (button press = 0 ms)**. Time-frequency decomposition (wavelet transform) of a single MEG sensor shows strong beta power (∼20 Hz) suppression and high gamma synchronization (∼80 Hz) both preceding and around movement onset (0 ms).

**Figure 2:**
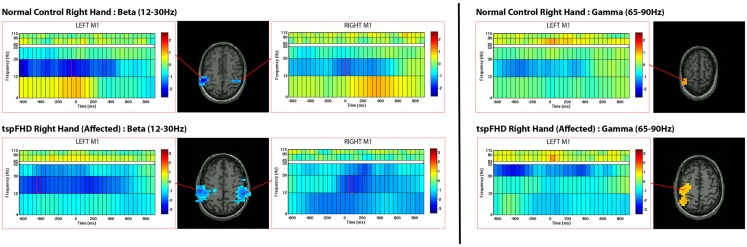
**Source reconstructions from single-subject motor task data in an exemplary healthy control (top row) and patient with tspFHD (bottom row)**. Changes in oscillatory activity are relative to a pre-stimulus baseline period and time-locked to movement onset (time = 0 ms). Significant decreases (in blue) in beta (12–30 Hz) power are identified over bilateral motor cortices in both subjects. Significant increases (in red) in high gamma (65–90 Hz) power are identified over similar regions of motor cortex.

**Figure 3:**
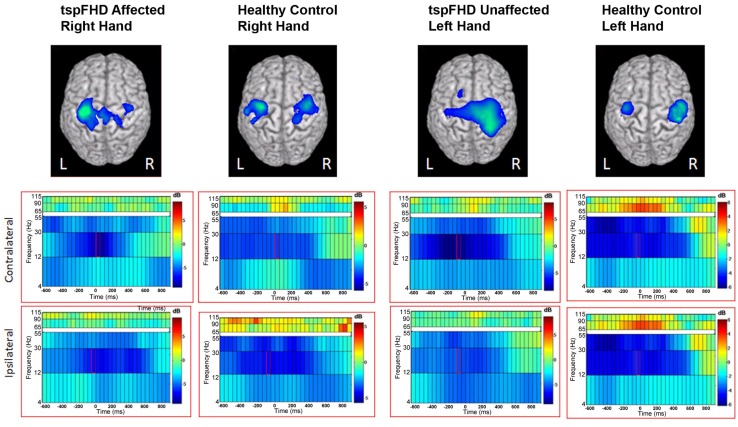
**Group data from the time-frequency analysis of beta band (12–30 Hz) activity**. Average reconstruction results for the self-paced button press superimposed on MNI template brains. The functional maps (one-sample *t*-test) are thresholded at *p* < 0.05 multiple comparisons correction (maximal statistic). The time-frequency spectrum displays 600 ms prior to the self-paced button press (time = 0 ms) through 1000 ms after the button press. Frequency bands are represented along the *y* axis. The peak activation in the contralateral and ipsilateral primary motor cortex is shown with the corresponding spectrograms below.

**Figure 4:**
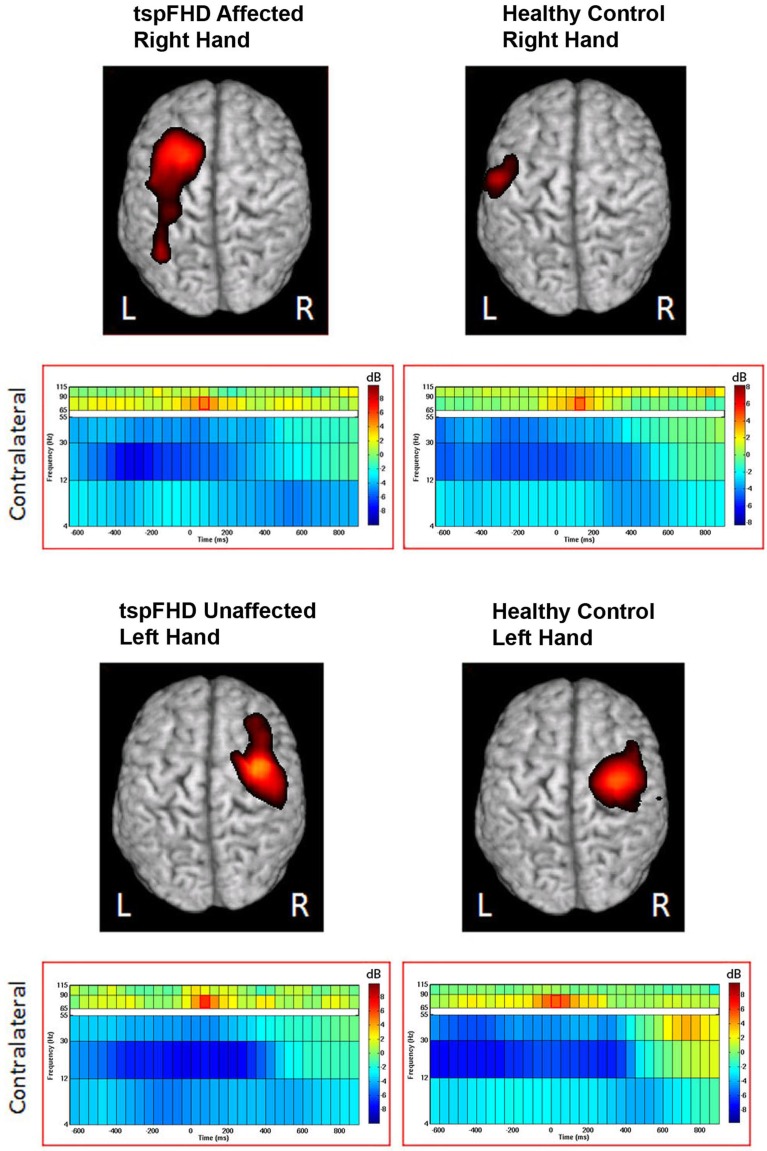
**Group data from the time-frequency analysis of high gamma band (65–90 Hz) activity**. Functional maps (one-sample *t*-test) are thresholded at *p* < 0.05 (maximal statistic). Peak activation in contralateral motor cortices are shown with the corresponding spectrograms below. Conventions as in Figure 2.

Time-frequency (wavelet) decompositions of single MEG sensor data from three healthy control subjects are shown in Figure 1. At the sensor-level, time-frequency analyses show that around button press (0 ms; Figure 1) significant reductions in beta (∼20 Hz) power and increases in high gamma (∼80 Hz) power occur over MEG channels contralateral to the hand being used (Figure 1). The MEG channel that shows the greatest change in power for these two frequency bands varies from subject to subject (Figure 1), making it difficult to localize the cortical origin of these changes in oscillatory power. Localized activity can be identified in the source space analyses, where both subjects with tspFHD and healthy controls demonstrated consistent beta band (12–30 Hz) power decreases bilaterally and high gamma (65–90 Hz) power increases contralaterally across the motor cortices (M1, PMC) around movement onset (0 ms; Figure 2). Significant (*p* < 0.05, FWE) reductions in beta power were identified over primary and pre-motor cortex bilaterally across groups (Figure 3). Significant (*p* < 0.005 uncorrected) increases in high gamma power were also identified over primary and pre-motor cortex around movement onset across both groups (Figure 4).

#### Group comparison: movements of the affected hand

**Figure 5:**
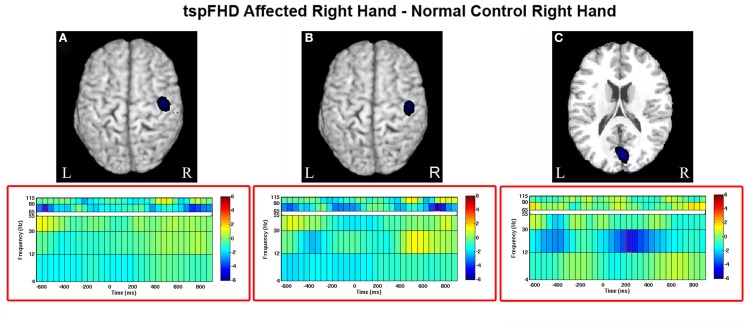
**Whole brain contrasts for movements of the affected hand**. Group comparisons (tspFHD > healthy control) for the self-paced button press superimposed on MNI template brains. In tspFHD, decreases increases in high gamma (65–90 Hz) activity over ipsilateral motor cortex occur prior to **(A)** and following **(B)** button press. In beta (12–30 Hz), increases in activity over visual cortex occur during button press **(C)**. All other conventions as in Figure 2.

Significant differences in oscillatory power between patients with tspFHD and healthy controls during movements of the right hand (affected in patients) was identified in the high gamma (65–90 Hz) band over sensorimotor cortex, for periods prior to (Figure 5A) and following (Figure 5B) hand movement. For time windows preceding the response with the affected hand (−575 ms), significant (*p* < 0.05, 1% FDR corrected) reductions in high gamma power were identified over the right post-central gyrus (MNI coordinates: 50, −15, 65; Figure 5A) in patients with tspFHD (mean high gamma power = −0.39) when compared to right hand movements of the healthy controls (mean high gamma power = 0.28). For time windows following the response with the affected hand (725 ms), significant (*p* < 0.05, 1% FDR corrected) reductions in high gamma power were also identified over the same region of ipsilateral sensorimotor cortex (MNI coordinates: 60, −10, 75; Figure 5B) in patients with tspFHD (mean high gamma power = −0.35) compared to healthy controls (mean high gamma power = 0.30). At the time of button press, no significant changes in high gamma power were identified over either ipsilateral (see 0 ms; Figures 5A,B) or contralateral sensorimotor cortex.

A significant (*p* < 0.05, 1% FDR corrected) difference between the two groups in beta power was seen over visual cortex during movements of the right (affected) hand (Figure 5C). In a period following button press (225 ms) beta power suppression over the cuneus of the occipital lobe (MNI coordinates: 50, 0, 35) was greater during movements of the right hand in patients with tspFHD (mean beta power = −0.46) compared to the same region in healthy controls (mean beta power = 0.26), indicating greater neural activity in this region in the patient group.

#### Group comparison: movements of the unaffected hand

**Figure 6:**
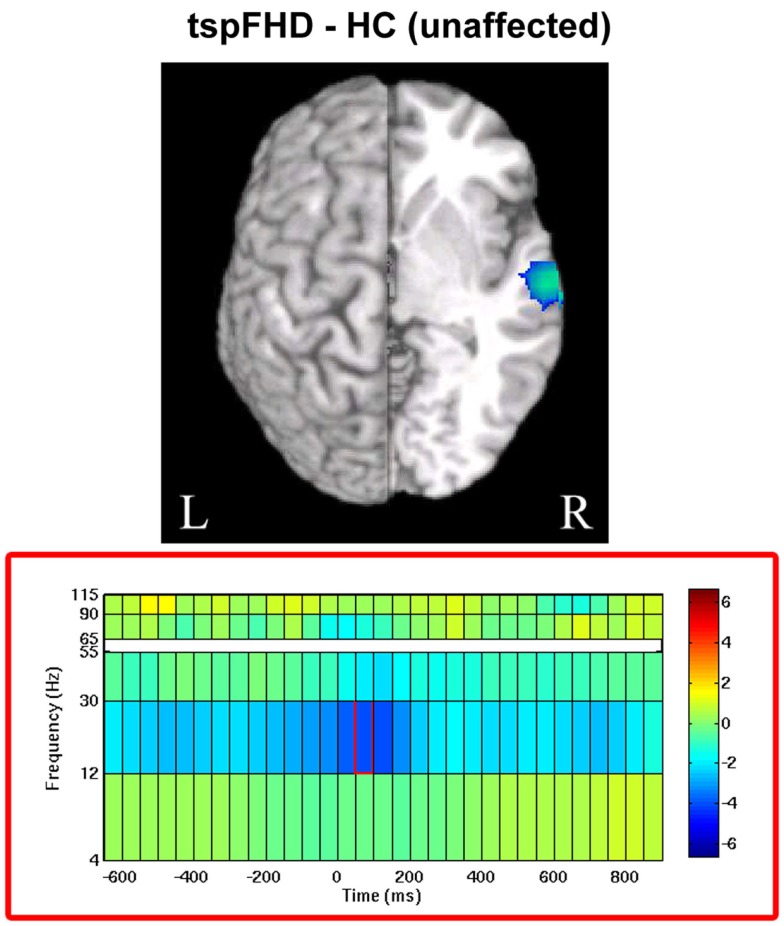
**Whole brain contrasts for movements of the unaffected hand**. In tspFHD, increases in beta (12–30 Hz) power suppression localize to secondary somatosensory cortex. All conventions as in Figure 2.

For movements of the left hand (unaffected in patients), a significant difference (*p* < 0.05, 1% FDR corrected) was identified between the two groups in beta power over secondary somatosensory cortex in the right hemisphere (MNI coordinates: 65, −15, 5; Figure 6). In the period following movement onset (75 ms), greater beta power suppression was observed during movements of the unaffected hand in patients with tspFHD (mean beta power = −0.68) relative to healthy controls (mean beta power = −0.19), indicating greater neural activity following button press. There were no significant differences between the unaffected left hand of subjects with tspFHD and the left hand of healthy controls in the high gamma band at any time point.

### Correlation and linear regression analyses

As in previous studies, when our tspFHD sample is compared to normative data available from healthy controls, the subjects with tspFHD demonstrated significant impairments in graphesthesia and stereognosis, longer digital reaction times, and a reduced strength ratio of the intrinsic to the extrinsic muscles of the hand (all *p* < 0.05). However, there were no statistically significant correlations between the clinical motor performance measures and either the latency or amplitude of the beta peak in either contra- or ipsilateral M1 during movements of either the affected or unaffected hand in tspFHD (all *p* > 0.05).

## Discussion

The present study used MEG to provide unique information about the spatial and temporal properties of cortical motor processing across the two hemispheres in subjects with tspFHD. There were clear problems in the timing and amplitude of activity in sensory and motor cortex in this population. Specifically, increases in oscillatory power were observed over either primary visual cortex or secondary somatosensory cortex during movements of the affected or unaffected hand, respectively. More importantly, decreased activity over ipsilateral M1 during movements of the affected digits in tspFHD occurred later and ended earlier when compared to healthy controls. These findings support the posit that the timing of activity in the motor system is impaired in tspFHD. This data is consistent with an overall observation of aberrant oscillatory power in tspFHD obtained from power analyses of EEG sensor data during both self-paced (Toro et al., 2000; Kristeva et al., 2005) and cued movement (Ruiz et al., 2009). It is likely that impairments in both cortical excitability and intracortical inhibition contribute to these aberrant patterns of activity we identify in our patient sample. The source localization provided by our own analyses indicate that separate cortical fields contribute differently to changes in oscillatory power in tspFHD, with beta power differences specific to sensory cortices, and high gamma power changes specific to motor cortex.

### Abnormal high gamma processing in ipsilateral primary motor cortex

Current theories of the cortical motor system in focal dystonia are based upon the assumption that center-surround systems of lateral inhibition (or “intracortical inhibition”; Sohn and Hallett, 2004; Stinear and Byblow, 2004) are impaired in tspFHD. If these mechanisms (which fine-tune M1 output in order to create discrete movements of the digits) are impaired in these patients, it could lead to the clinical expression of the disorder (e.g., an inability to restrain abnormal contractions). In our study, although changes in M1 high gamma activity around *the time of the button press* did not differ in response magnitude between patients and controls for either hemisphere (Figure 5), robust differences in high gamma activity over ipsilateral M1 were identified in time windows *prior to and following* the button press. These findings suggest a failure to activate the motor system appropriately in tspFHD that occurs when these individuals are using the affected hand (Figures 5A,B). These findings further expand on the observation of impaired intracortical inhibition in tspFHD by demonstrating that both movement initiation and inhibition is compromised in these patients.

It has been previously suggested that not only do processing abnormalities exist in the hemisphere ipsilateral to the affected hand in tspFHD, but that these impairments play a key role in the manifestation of the disorder. Topographic disorganization in the ipsilateral somatosensory cortices in tspFHD coincides with the magnitude of clinical impairment in these patients (Meunier et al., 2001). Coupling between ipsilateral and contralateral sensorimotor cortices is aberrantly increased in tspFHD (Butz et al., 2006). Several TMS studies have suggested that compromised ipsilateral processing is related to impairments in cortical inhibition (Sommer et al., 2002) through the loss of regulated transcallosal transmission of M1 (Niehaus et al., 2001) particularly in patients with unilateral symptom presentation (Meunier et al., 2001; Nelson et al., 2010).

Although this may appear conflicting with fMRI and PET studies of motor function in tspFHD, where activity increases over the motor cortex during simple movements (Blood et al., 2004; Obermann et al., 2008) and task-specific behaviors (Pujol et al., 2000), our data indicates that changes in cortical activity in focal dystonia are not specific to movement onset. As the changes we report in the present study occur on a short time scale (∼600 ms prior to and following movement), reduced cortical activity in ipsilateral M1 may reflect aberrant initiation and inhibition in motor cortex more specifically, and not movement execution *per se*. Since we were able to observe changes in cortical oscillations even prior to the movement on the millisecond timescale, the likelihood of somatosensory interference confounding our results are unlikely. Significant increases in high gamma activity are specific to the motor act and not due to somatosensory feedback, as they are present only during active movement and not passive finger flexion (Muthukumaraswamy, 2010).

### Abnormal beta processing in sensory cortex

In contrast to changes in high gamma activity identified in the tspFHD group over ipsilateral motor cortex, differences in the beta band between the two groups were primarily identified in sensory cortices. Specifically, increased cortical activity was observed over the cuneus during movements of the affected hand in tspFHD patients in the time period following button press onset (Figure 5C). It is possible that increased activity in visual cortex is related to a greater reliance on other sensory modalities and compensation strategies, such as visualization of the movement. Patients with tspFHD are behaviorally impaired on mental rotation tasks involving the hands but not the feet (Fiorio et al., 2006). Furthermore, BOLD fMRI studies on tspFHD have reported increased activity in visual cortex during tactile discrimination tasks, indicating a greater reliance on this sensory modality during manual behavior (Peller et al., 2006). For movements of the unaffected hand, increases in cortical activity (beta power suppression) in the contralateral hemisphere over S2 are present in patients with tspFHD around button press (Figure 6). Abnormal activation and reorganization of secondary somatosensory cortex is known to exist in patients with tspFHD (Butterworth et al., 2003). It is possible that this heightened beta activity over somatosensory cortex during movements of a less impacted effector acts as a precursor to symptoms of tspFHD.

### Clinical implications

Behavioral interventions rooted in principles of neuroscience have been successful in the treatment of focal dystonia (see Hinkley et al., 2009 for a review). Therefore, one can speculate how the information we derive from our MEG analyses might be used to design novel treatment interventions for dystonia. For example, the poor timing within activation of ipsilateral primary motor cortex during movement of the affected hands suggests a failure of being able to intrinsically select the affected musculature during a task. Furthermore, increased activity in the visual system during movement suggests a greater reliance upon other sensory mechanisms, such as imagery, to guide motor control. Biofeedback, which provides auditory and visual information to control inappropriate muscle contractions, may be a successful component of retraining through rehabilitating this aberrant timing in the motor system. This method has been reported as being effective in retraining patients with writer’s cramp (Cottraux et al., 1983; Baur et al., 2009). Although it is not clear how to specifically activate and retrain the ipsilateral pathway in tspFHD, the remediation of ipsilateral cortical activity through bilateral motor training has been shown to be particularly effective in the treatment of stroke-induced hemiparesis (Stewart et al., 2006; McCombe-Waller et al., 2008). In tspFHD, bilateral practice as a potential treatment may help activate an ipsilateral motor pathway and increase inhibition between the motor cortices, therefore regulating oscillatory power.

### Limitations of the study

Although we report robust differences in activity between patients and controls, in spite of the small subject size, it is possible that other differences in oscillatory activity between the tspFHD and healthy control groups failed to reach statistical significance due to the small sample size (11 participants in each group). Unfortunately, given the low prevalence of this disorder and the restrictions placed on participation (claustrophobia, metallic implants) it is difficult to enroll a large number of subjects in an imaging study from one local area. Cross-site collaborations and standardization of imaging protocols are necessary to strengthen sample sizes for uncommon neurological disorders like tspFHD. It is possible that an absence of correlations between the behavioral and MEG data might be due to the patients executing a non-dystonic task, even though it would not be possible to design a common task that would elicit these symptoms across such a heterogeneous sample (Table 1). Although it could be argued that the differences in motor activity we observe in the tspFHD group are due to changes in cortical excitability induced by botulinum toxin treatments, of the three patients that did receive these injections (Table 1) only one dose was administered, and no effect of the treatment was observed. It is also possible that subtle muscle contractions in the patient group during the task contributed to noise in the dataset and fine movement parameters not measured by our equipment (such as the period between the initial movement onset to button press) influence the changes in MEG signal we observe here. Future MEG studies will need concurrent recording of MEG and EMG to detect these subtle abnormalities in flexion and extension. Further, it is also conceivable that currently available ordinal rather than quantitative clinical measures of dystonia are not sensitive or specific enough to accurately represent the severity of the movement dysfunction in tspFHD (Spector and Branforbrener, 2007). Poor sensitivity may account for a lack of correlation between aberrant temporal and spatial motor responses in the present study.

## Conclusion

The properties of movement preparation and execution in sensorimotor cortex in patients with tspFHD are far more complex than just changes in oscillatory power. Although it is difficult to discern if the abnormal responses identified in this study predispose an individual to dystonia or are the net consequence of aberrant posturing, data from neuroimaging studies like ours provide a neurophysiological foundation to guide clinical intervention strategies. Nonetheless, these findings underscore the need for a greater understanding of complex oscillatory dynamics in order to both test the efficacy of existing treatments (like biofeedback) and to develop novel therapeutics (for example, bilateral sensorimotor retraining) for task-specific dystonia.

## Conflict of Interest Statement

The authors declare that the research was conducted in the absence of any commercial or financial relationships that could be construed as a potential conflict of interest.

## Footnotes

^1^http://nutmeg.berkeley.edu

^2^www.qb3.org

^3^http://imaging.mrc-cbu.cam.ac.uk/downloads/MNI2tal/mni2tal.m

^4^http://www.talairach.org/daemon.html
